# Supplementary material for: Evolution of gene dosage on the Z-chromosome of schistosome parasites
Source: eLife. 2018 Jul 25;7:e35684. doi: 10.7554/eLife.35684 (PMC6089595; doi:10.7554/eLife.35684)
Supplement: Supplementary file 1. — Comparison of female:male ratios of expression (F:M) using microarray and proteomics (Supplementary Table 2) [file elife-35684-supp1.docx]

**Supplementary Table 1. Comparison of the ratio of expression between the Z-linked and autosomal genes (Z:A), and comparison of expression between males and females (F:M) in the different species, stages and methods.**

The ratios “without ancestry” correspond to those of Figure 2 and the Z-specific region corresponds to S0+S1jap (in *S. japonicum*) or S0+S1mans (in *S. mansoni*). The ratios “with ancestry” use expression values normalized by those of the other species, and correspond to Figure 3. The Z-specific region corresponds in this case to S1jap (in *S. japonicum*) or S1mans (in *S. mansoni*). Level of significance (Wilcoxon rank sum test with continuity correction): **P-value*<0.05, ***P-value*<0.001, ****P-value*<0.0001, N.S. *P-value*>0.05.

|  | *S. japonicum* whole worm | | | | *S. mansoni* whole worm | | | |
| --- | --- | --- | --- | --- | --- | --- | --- | --- |
|  | Without ancestry | | With ancestry | | Without ancestry | | With ancestry | |
|  | Schistosomula  *Fig 2.A.* | Adult  *Fig 2.B.* | Schistosomula  *Fig 3.A.* | Adult  *Fig 3.B.* | Schistosomula  *Fig 2.C.* | Adult  *Fig 2.D.* | Schistosomula  *Fig. 3.C.* | Adult  *Fig 3.D.* |
| Female Z:AA | 0.81  * | 0.81  * | 0.80  * | 0.68  * | 0.85  * | 0.73  ** | 0.83  * | 0.73  N.S. |
| Male ZZ:AA | 1.46  *** | 1.25  * | 1.24  * | 0.98  N.S. | 1.37  *** | 1.35  * | 1.22  * | 1.35  * |
| (F:M)Z | 0.58  *** | 0.62  ** | 0.63  * | 0.69  * | 0.63  *** | 0.62  *** | 0.70  * | 0.54  ** |
| (F:M)A | 1.04  N.S. | 1.12  * | 0.97  N.S. | 0.99  N.S. | 1.05  N.S. | 1.15  N.S. | 1.03  N.S. | 1.01  N.S. |
| (F:M)Z/(F:M)A | 0.56  *** | 0.55  *** | 0.65  *** | 0.69  *** | 0.60  *** | 0.54  *** | 0.68  *** | 0.54  *** |

**Supplementary Table 2. Comparison of female:male ratios of expression (F:M) using microarrays and proteomics.** Z refers to Z-linked genes, A to autosomal genes.

|  | *Schistosoma mansoni* | | | |
| --- | --- | --- | --- | --- |
|  | Heads | | Gonads | |
|  | Microarrays *Not shown.* | Proteomics *Fig 4.A.* | Microarrays *Not shown.* | Proteomics *Fig 4.B.* |
| (F:M)Z | 0.67 | 0.68 | 0.74 | 0.78 |
| (F:M)A | 1.00 | 0.92 | 1.17 | 0.99 |
| (F:M)Z/(F:M)A | 0.66 * | 0.75 *** | 0.63 *** | 0.79 *** |
